# Supplementary material for: Differentiation and Interconnection of the Bacterial Community Associated with Silene nigrescens Along the Soil-To-Plant Continuum in the Sub-Nival Belt of the Qiangyong Glacier
Source: Plants (Basel). 2025 Apr 11;14(8):1190. doi: 10.3390/plants14081190 (PMC12030249; doi:10.3390/plants14081190)
Supplement: Supplementary file 1 [file plants-14-01190-s001.zip › plants-3521059-supplementary.pdf]

Title Differentiation and connection of the bacterial community associated with *Silene nigrescens* along the soil-to-plant continuum in the sub-nival belt of the Qiangyong Glacier

Authors

Wangchen Sonam, Yongqin Liu, Luming Ren

This Supplementary File includes Tables S1-S8 and Figures S1-S2:

Table S1 The geographic information of the *Silene nigrescens* (*S.nigrescens*) was collected from the sub-nival belt of the Qiangyong glacier.

Table S2 The differences in levels and ratios of total nitrogen (TN), total phosphorus (TP), and total potassium (TK) among soil, roots, and leaves. Different lowercase letters denote significant differences in the levels and ratios of total nitrogen (TN), total phosphorus (TP), and total potassium (TK) among soil, roots, and leaves, as identified by the Wilcoxon Test with a significance level of $P < 0.05$.

Table S3. The correlation between the levels and ratios of TN (total nitrogen), TP (total phosphorus), and TK (total potassium) and the bacterial community composition was determined using the Mantel test.

Table S4. Keystone nodes in the co-occurrence networks of the bacterial communities associated with the rhizosphere soil, root endosphere, and leaf endosphere.

Table S5. Keystone nodes in the co-occurrence networks of bacterial community, encompassing all taxa present in rhizosphere soil, root endosphere, and leaf endosphere.

Table S6. Spearman correlation between alpha diversity and the average variation degree (AVD) of the bacterial community. ***$P < 0.001$.

Table S7. Amplicon sequence variants (ASVs) that are common among the rhizosphere soil, root endosphere, and leaf endosphere.

Table S8. Key topological properties of bacterial co-occurrence networks, encompassing all taxa within the rhizosphere soil, leaf endosphere, and root endosphere.

Figure S1. The location of the Qiangyong glacier (a, b), and the *Silene nigrescens* (*S.nigrescens*)

growing in the Qiangyong glacier terminus (c).

Figure S2. Rarefaction curves of ASV richness and Shannon diversity.

Table S1 The geographic information of the *Silene nigrescens* (*S.nigrescens*) was collected from the sub-nival belt of the Qiangyong glacier.

Sample ID	Longitude (N)	Latitude (E)	Elevation (m)
*S.nigrescens*_1	28°52′36.31″	90°13′29.74″	5110
*S.nigrescens*_2	28°52′37.00″	90°13′30.63″	5091
*S.nigrescens*_3	28°52'40.88"	90°13'35.42"	5072
*S.nigrescens*_4	28°52′41.71″	90°13′29.97″	5061
*S.nigrescens*_5	28°52′39.32″	90°13′30.35″	5059
*S.nigrescens*_6	28°52'42.37"	90°13'37.12"	5035
*S.nigrescens*_7	28°52'47.53"	90°13'33.72"	4995
*S.nigrescens*_8	28°52'49.25"	90°13'33.38"	4978
*S.nigrescens*_9	28°52'49.45"	90°13'33.35"	4974
*S.nigrescens*_10	28°52'51.05"	90°13'33.48"	4962

Table S2 The differences in levels and ratios of total nitrogen (TN), total phosphorus (TP), and total potassium (TK) among soil, roots, and leaves. Different lowercase letters denote significant differences in the levels and ratios of total nitrogen (TN), total phosphorus (TP), and total potassium (TK) among soil, roots, and leaves, as identified by the Wilcoxon Test with a significance level of $P < 0.05$.

	Soils	Roots	leaves
TN	0.339 ± 0.097c	5.786 ± 0.095b	27.491 ± 1.870a
TP	0.639 ± 0.041b	0.528 ± 0.035a	0.534 ± 0.217a
TK	13.274 ± 0.3623a	4.561 ± 0.369b	2.886 ± 0.252c
TN:TP:TK	0.040 ± 0.012c	2.422 ± 0.207b	20.379 ± 7.363a
TN:TP	0.536 ± 0.166c	10.996 ± 0.563b	59.142 ± 23.093a
TN:TK	0.025 ± 0.007c	1.276 ± 0.111b	9.559 ± 0.694a
TP:TK	0.048 ± 0.003c	0.116 ± 0.013b	0.188 ± 0.084a

Table S3. The correlation between the levels and ratios of TN (total nitrogen), TP (total phosphorus), and TK (total potassium) and the bacterial community composition was determined using the Mantel test.

Variable	Mantel test (*r* statistics)	*P*-value
TN	0.599	0.0004
TP	0.135	0.0766
TK	0.692	0.0001
TN:TP:TK	0.459	0.0011
TN:TP	0.445	0.0005
TN:TK	0.517	0.0007
TP:TK	0.166	0.0557

Table S4. Keystone nodes in the co-occurrence networks of the bacterial communities associated with the rhizosphere soil, root endosphere, and leaf endosphere.

ASV ID	Within module connectivities	Among module connectivities	Degree	Modularity	Type	Taxonomy	Plant compartment niche
ASV143	-2.28	0.75	2	7	Connectors	*Pseudomonas*	Rhizosphere soil
ASV245	-0.30	0.75	2	3	Connectors	Rhizobiales	Rhizosphere soil
ASV134	-0.30	0.75	2	3	Connectors	Acetobacteraceae	Rhizosphere soil
ASV30	-1.40	0.75	2	19	Connectors	*Devosia*	Rhizosphere soil
ASV305	-0.54	0.75	2	15	Connectors	Oxalobacteraceae	Rhizosphere soil
ASV416	-0.59	0.75	2	20	Connectors	*Craurococcus-Caldovatus*	Rhizosphere soil
ASV94	-1.09	0.69	4	19	Connectors	*Brevundimonas*	Rhizosphere soil
ASV135	-0.54	0.75	2	15	Connectors	*Conexibacter*	Rhizosphere soil
ASV104	-0.50	0.75	2	14	Connectors	*Mycobacterium*	Rhizosphere soil
ASV75	-1.40	0.78	3	19	Connectors	*Solirubrobacter*	Rhizosphere soil
ASV81	-0.60	0.75	2	2	Connectors	*Actinoplanes*	Rhizosphere soil
ASV522	-0.59	0.75	2	20	Connectors	*Rathayibacter*	Rhizosphere soil
ASV91	-0.67	0.69	4	12	Connectors	*Lautropia*	Rhizosphere soil
ASV415	-0.59	0.75	2	20	Connectors	*Sphingomonas*	Rhizosphere soil
ASV301	-0.60	0.75	2	2	Connectors	Gaiellales	Rhizosphere soil
ASV28	-0.50	0.75	2	14	Connectors	*Pseudonocardia*	Rhizosphere soil
ASV64	-1.98	0.75	2	11	Connectors	*Allorhizobium-Neorhizobium-Pararhizobium-Rhizobium*	Rhizosphere soil
ASV802	-0.50	0.75	2	14	Connectors	Hyphomicrobiaceae	Rhizosphere soil
ASV605	-0.54	0.75	2	15	Connectors	Frankiales	Rhizosphere soil
ASV390	-0.54	0.75	2	15	Connectors	*TK10*	Rhizosphere soil
ASV76	-1.26	0.75	2	17	Connectors	*Pseudomonas*	Rhizosphere soil
ASV348	-0.50	0.75	2	14	Connectors	*Solirubrobacter*	Rhizosphere soil
ASV43	-0.30	0.78	3	3	Connectors	*Lechevalieria*	Rhizosphere soil
ASV147	-1.34	0.75	2	12	Connectors	*Kineosporia*	Rhizosphere soil
ASV266	0.00	0.6875	4	22	Connectors	Beijerinckiaceae	Rhizosphere soil

ASV82	-1.12	0.78	3	10	Connectors	*IMCC26256*	Rhizosphere soil
ASV11	0.00	0.75	2	24	Connectors	*Actinoplanes*	Rhizosphere soil
ASV339	-0.99	0.75	2	1	Connectors	*Geodermatophilus*	Rhizosphere soil
ASV127	-0.60	0.75	2	2	Connectors	*Pedomicrobium*	Rhizosphere soil
ASV79	-1.09	0.68	5	19	Connectors	*Solirubrobacter*	Rhizosphere soil
ASV535	-1.401	0.78	3	19	Connectors	*Clavibacter*	Rhizosphere soil
ASV89	0.00	0.75	2	10	Connectors	*Sphingomonas*	Rhizosphere soil
ASV24	-0.99	0.75	2	1	Connectors	*Pseudonocardia*	Rhizosphere soil
ASV8	-0.60	0.75	2	2	Connectors	*Pseudomonas*	Rhizosphere soil
ASV387	-0.60	0.75	2	2	Connectors	*Nakamurella*	Rhizosphere soil
ASV208	-0.30	0.75	2	3	Connectors	*Microbacterium*	Rhizosphere soil
ASV197	0.00	0.75	2	24	Connectors	*Geodermatophilus*	Rhizosphere soil
ASV315	-0.50	0.75	2	14	Connectors	*Reyranella*	Rhizosphere soil
ASV341	-0.54	0.75	2	15	Connectors	*Sphingomonas*	Rhizosphere soil
ASV120	-0.60	0.75	2	2	Connectors	*Bradyrhizobium*	Rhizosphere soil
ASV40	-0.30	0.75	2	3	Connectors	*Nocardioides*	Rhizosphere soil
ASV85	0.00	0.75	2	10	Connectors	Microtrichales	Rhizosphere soil
ASV201	-0.60	0.75	2	2	Connectors	*Geodermatophilus*	Rhizosphere soil
ASV117	-1.26	0.75	2	17	Connectors	Rhizobiales	Rhizosphere soil
ASV318	-1.35	0.75	2	12	Connectors	*Devosia*	Rhizosphere soil
ASV213	-1.40	0.75	2	21	Connectors	*Blastococcus*	Rhizosphere soil
ASV152	-1.26	0.75	2	17	Connectors	Frankiales	Rhizosphere soil
ASV90	-1.35	0.69	4	12	Connectors	*Nakamurella*	Rhizosphere soil
ASV389	-0.50	0.75	2	14	Connectors	*Methylobacterium-Methylorubrum*	Rhizosphere soil
ASV25	-0.60	0.75	2	2	Connectors	*Devosia*	Rhizosphere soil
ASV22	-0.60	0.78	3	2	Connectors	Frankiales	Rhizosphere soil
ASV292	-0.30	0.75	2	3	Connectors	*Pseudonocardia*	Rhizosphere soil
ASV87	0.00	0.75	2	24	Connectors	*Pseudonocardia*	Rhizosphere soil
ASV216	-0.46	0.6875	4	1	Connectors	*Nocardioides*	Rhizosphere soil
ASV194	0.40	0.68	5	2	Connectors	*Conexibacter*	Rhizosphere soil
ASV157	-0.60	0.75	2	2	Connectors	*67-14*	Rhizosphere soil
ASV141	-1.79	0.75	2	7	Connectors	*Microbacterium*	Rhizosphere soil
ASV53	-1.09	0.69	4	19	Connectors	*Blastococcus*	Rhizosphere soil
ASV349	2.80	0.21	9	1	Module hubs	Rhizobiales	Rhizosphere soil
ASV184	-0.60	0.75	2	2	Connectors	*Modestobacter*	Rhizosphere soil
ASV291	-0.50	0.75	2	14	Connectors	*Quadrisphaera*	Rhizosphere soil
ASV173	-0.59	0.75	2	20	Connectors	*Microvirga*	Rhizosphere soil
ASV70	-2.02	0.78	3	6	Connectors	*Blastococcus*	Rhizosphere soil
ASV444	-1.12	0.75	2	10	Connectors	*Rhodoplanes*	Rhizosphere soil
ASV116	-0.99	0.75	2	1	Connectors	*Mycobacterium*	Rhizosphere soil
ASV394	-0.54	0.75	2	15	Connectors	*Sulfurirhabdus*	Rhizosphere soil
ASV733	-0.59	0.75	2	20	Connectors	*Phycicoccus*	Rhizosphere soil
ASV63	0.87	0.75	2	6	Connectors	*Saccharothrix*	Root endosphere

ASV32	-1.62	0.75	2	3	Connectors	*Delftia*	Root endosphere
ASV150	-0.43	0.75	2	4	Connectors	*Aureimonas*	Root endosphere
ASV28	-0.43	0.75	2	4	Connectors	*Pseudonocardia*	Root endosphere
ASV199	1.15	0.75	2	13	Connectors	*Pseudomonas*	Root endosphere
ASV161	0.00	0.75	2	12	Connectors	*Allorhizobium-Neorhizobium-Pararhizobium-Rhizobium*	Root endosphere
ASV80	-0.73	0.75	2	7	Connectors	Micrococcaceae	Root endosphere
ASV316	-0.43	0.75	2	4	Connectors	*Sphingobium*	Root endosphere
ASV370	-0.43	0.75	2	4	Connectors	*Asticcacaulis*	Root endosphere
ASV35	-0.43	0.75	2	4	Connectors	Stappiaceae	Root endosphere
ASV18	-1.62	0.75	2	3	Connectors	*Bosea*	Root endosphere
ASV8	1.15	0.75	2	15	Connectors	*Pseudomonas*	Root endosphere
ASV1	1.15	0.75	2	14	Connectors	*Pseudomonas*	Root endosphere
ASV4	0.00	0.75	2	12	Connectors	*Conyzicola*	Root endosphere
ASV40	0.87	0.75	2	6	Connectors	*Nocardioides*	Root endosphere
ASV13	-0.45	0.75	2	8	Connectors	*Pseudomonas*	Root endosphere
ASV23	-0.73	0.75	2	7	Connectors	*Gaiella*	Root endosphere
ASV513	-0.73	0.75	2	7	Connectors	*Sphingomonas*	Root endosphere
ASV15	-0.43	0.75	2	4	Connectors	*Tardiphaga*	Root endosphere
ASV34	-0.43	0.75	2	4	Connectors	*Mesorhizobium*	Root endosphere
ASV55	-0.43	0.75	2	4	Connectors	*Altererythrobacter*	Root endosphere
ASV36	-0.71	0.69	4	1	Connectors	*Brevundimonas*	Root endosphere
ASV118	-0.87	0.69	4	3	Connectors	*Acidovorax*	Root endosphere
ASV52	-0.43	0.75	2	4	Connectors	*Pseudolabrys*	Root endosphere
ASV70	0.00	0.75	2	12	Connectors	*Blastococcus*	Root endosphere
ASV112	-0.43	0.75	2	4	Connectors	*Aminobacter*	Root endosphere
ASV49	-0.39	0.75	2	1	Connectors	*Pseudomonas*	Leaf endosphere
ASV14	-0.39	0.75	2	1	Connectors	*Pseudomonas*	Leaf endosphere
ASV140	1.15	0.75	2	3	Connectors	*Pseudomonas*	Leaf endosphere
ASV2	-0.39	0.75	2	1	Connectors	*Pseudomonas*	Leaf endosphere

Table S5. Keystone nodes in the co-occurrence networks of bacterial community, encompassing all taxa present in rhizosphere soil, root endosphere, and leaf endosphere.

ASV ID	Within module connectivities	Among module connectivities	Degree	Modularity	Type	Taxonomy	Counts
ASV257	-1.27	0.75	2	4	Connectors	Acetobacteraceae	1
ASV185	1.15	0.75	2	8	Connectors	Beijerinckiaceae	2
ASV266	-0.14	0.69	6	4	Connectors	Beijerinckiaceae	
ASV211	-0.70	0.68	5	1	Connectors	Microbacteriaceae	1
ASV227	-0.88	0.65	11	3	Connectors	Rhizobiales	1
ASV372	-0.73	0.75	2	7	Connectors	*67-14*	2
ASV313	-0.14	0.63	7	4	Connectors	*67-14*	
ASV55	-0.99	0.75	2	2	Connectors	*Altererythrobacter*	
ASV119	-0.86	0.78	3	6	Connectors	*Arthrobacter*	2

ASV295	-1.24	0.75	2	3	Connectors	*Arthrobacter*	
ASV249	-0.96	0.78	3	1	Connectors	*Aureimonas*	2
ASV261	-0.19	0.76	5	6	Connectors	*Aureimonas*	
ASV56	-0.99	0.75	2	2	Connectors	*Brevundimonas*	1
ASV45	-0.73	0.75	2	7	Connectors	*Conexibacter*	
ASV135	-0.89	0.75	2	4	Connectors	*Conexibacter*	3
ASV214	-0.70	0.71	7	1	Connectors	*Conexibacter*	
ASV77	-1.27	0.75	2	4	Connectors	*Conyzicola*	1
ASV151	-0.14	0.64	6	4	Connectors	*Cryobacterium*	1
ASV32	1.15	0.75	2	9	Connectors	*Delftia*	1
ASV162	-0.96	0.78	3	1	Connectors	*Devosia*	2
ASV318	-1.22	0.75	2	1	Connectors	*Devosia*	
ASV252	-0.14	0.63	7	4	Connectors	*Herbaspirillum*	1
ASV82	-1.00	0.64	6	3	Connectors	*IMCC26256*	1
ASV99	-0.96	0.81	4	1	Connectors	*Mesorhizobium*	1
ASV296	-0.89	0.78	3	4	Connectors	*Methylobacterium-Methylorubrum*	1
ASV37	-0.99	0.75	2	2	Connectors	*Microbacterium*	1
ASV184	1.15	0.81	4	5	Connectors	*Modestobacter*	1
ASV126	-0.96	0.69	4	1	Connectors	*Mycobacterium*	
ASV347	-0.73	0.75	2	7	Connectors	*Mycobacterium*	4
ASV29	-0.58	0.75	2	8	Connectors	*Mycobacterium*	
ASV186	-0.99	0.75	2	2	Connectors	*Mycobacterium*	
ASV319	-1.24	0.78	3	3	Connectors	*Nocardioides*	1
ASV69	-0.99	0.78	3	2	Connectors	*Phyllobacterium*	1
ASV253	-0.14	0.64	6	4	Connectors	*Pseudarthrobacter*	2
ASV335	-0.14	0.64	6	4	Connectors	*Pseudarthrobacter*	
ASV52	-0.99	0.75	2	2	Connectors	*Pseudolabrys*	1
ASV6	-0.86	0.75	2	6	Connectors	*Pseudomonas*	
ASV49	-0.86	0.75	2	6	Connectors	*Pseudomonas*	
ASV3	-0.86	0.75	2	6	Connectors	*Pseudomonas*	5
ASV1	0.48	0.64	6	6	Connectors	*Pseudomonas*	
ASV13	-0.86	0.75	2	6	Connectors	*Pseudomonas*	
ASV165	-0.52	0.69	4	4	Connectors	*Pseudonocardia*	2
ASV292	-1.24	0.75	2	3	Connectors	*Pseudonocardia*	
ASV290	-1.12	0.69	4	3	Connectors	*Pseudoxanthomonas*	1
ASV269	-0.43	0.64	6	1	Connectors	*Rhodococcus*	1
ASV206	-0.14	0.64	6	4	Connectors	*Roseococcus*	1
ASV209	0.09	0.65	10	1	Connectors	*Sphingomonas*	2
ASV89	-0.17	0.63	7	1	Connectors	*Sphingomonas*	
ASV242	-0.89	0.75	2	4	Connectors	*Thiobacillus*	1
ASV229	-0.14	0.64	6	4	Connectors	*Variovorax*	1

ASV152	-0.58	0.75	2	5	Connectors	Frankiales	2
ASV200	-0.89	0.75	2	4	Connectors	Frankiales	

Table S6. Spearman correlation between alpha diversity and the average variation degree (AVD) of the bacterial community. ***$P < 0.001$.

	AVD	Richness	Shannon	Pielou	Chao1	ACE
AVD		***	***	***	***	***
Richness	0.970		***	***	***	***
Shannon	0.946	0.949		***	***	***
Pielou	0.909	0.911	0.985		***	***
Chao1	0.970	1.000	0.949	0.911		***
ACE	0.968	0.999	0.946	0.907	0.999	

Table S7. Amplicon sequence variants (ASVs) that are common among the rhizosphere soil, root endosphere, and leaf endosphere.

ASV ID	Taxonomy	Count
The ASVs shared among the rhizosphere soil, root endosphere, and leaf endosphere		
ASV198	*0319-7L14*	1
ASV21	*Allorhizobium-Neorhizobium-Pararhizobium-Rhizobium*	1
ASV88, ASV120	*Bradyrhizobium*	2
ASV94, ASV155	*Brevundimonas*	2
ASV174	*Clavibacter*	1
ASV4	*Conyzicola*	1
ASV30	*Devosia*	1
ASV95	*Escherichia-Shigella*	1
ASV23, ASV102	*Gaiella*	2
ASV82, ASV108, ASV330	*IMCC26256*	3
ASV141	*Microbacterium*	1
ASV126	*Mycobacterium*	1
ASV90	*Nakamurella*	1
ASV40, ASV47, ASV66	*Nocardioides*	3
ASV69	*Phyllobacterium*	1
ASV44, ASV65, ASV73	*Pseudarthrobacter*	3
ASV395	*Pseudolabrys*	1
ASV1, ASV2, ASV3, ASV5, ASV6, ASV9, ASV13, ASV14, ASV16, ASV17, ASV76, ASV93	*Pseudomonas*	12
ASV932	*Rubrobacter*	1
ASV79	*Solirubrobacter*	1

ASV89, ASV101	*Sphingomonas*	2
ASV96	*Stenotrophomonas*	1
ASV22	Frankiales	1
ASV57	Gemmatimonadaceae	1
ASV117	Rhizobiales	1
ASV139	Acetobacteraceae	1
ASV273	Actinomarinales	1
ASV285, ASV42	Microbacteriaceae	2
ASV10	*Variovorax*	1
ASV35	Stappiaceae	1
ASV54	Sporichthyaceae	1
ASV80	Micrococcaceae	1
ASV85	Microtrichales	1
ASV353	Propionibacteriaceae	1
The ASVs shared between the rhizosphere soil and root endosphere		
ASV198, ASV410	*0319-7L14*	2
ASV423, ASV456, ASV485	*67-14*	3
ASV359, ASV644	*Actinophytocola*	2
ASV11	*Actinoplanes*	1
ASV21, ASV33, ASV64, ASV161, ASV195, ASV281, ASV308, ASV486, ASV547, ASV623, ASV679	*Allorhizobium-Neorhizobium-Pararhizobium-Rhizobium*	11
ASV517	*Amaricoccus*	1
ASV112, ASV512	*Aminobacter*	2
ASV260	*Amycolatopsis*	1
ASV403	*Ancylobacter*	1
ASV119, ASV166, ASV295	*Arthrobacter*	3
ASV137, ASV150, ASV191, ASV249, ASV261	*Aureimonas*	5
ASV653, ASV690	*Bacillus*	2
ASV27, ASV50, ASV70, ASV218	*Blastococcus*	4
ASV18, ASV202	*Bosea*	2
ASV88, ASV120, ASV175	*Bradyrhizobium*	3
ASV56, ASV94, ASV155	*Brevundimonas*	3
ASV721	*CL500-29_marine_group*	1
ASV174	*Clavibacter*	1
ASV677	*Clostridium_sensu_stricto_13*	1
ASV194, ASV651	*Conexibacter*	2
ASV4, ASV680	*Conyzicola*	2
ASV181	*D05-2*	1
ASV25, ASV30, ASV162, ASV309, ASV1240	*Devosia*	5
ASV95	*Escherichia-Shigella*	1
ASV23, ASV102, ASV231	*Gaiella*	3
ASV160	*Geodermatophilus*	1
ASV164	*Hyphomicrobium*	1
ASV358	*Ilumatobacter*	1

ASV82, ASV108, ASV330, ASV376, ASV763	*IMCC26256*	5
ASV147	*Kineosporia*	1
ASV207	*Kocuria*	1
ASV436	*Kribbella*	1
ASV91	*Lautropia*	1
ASV43	*Lechevalieria*	1
ASV310	*Leifsonia*	1
ASV204	*Marmoricola*	1
ASV106	*MB-A2-108*	1
ASV34, ASV99	*Mesorhizobium*	2
ASV37, ASV141, ASV652	*Microbacterium*	3
ASV357, ASV573	*Micromonospora*	2
ASV122, ASV173	*Microvirga*	2
ASV232, ASV346	*Modestobacter*	2
ASV29, ASV104, ASV126, ASV186, ASV324, ASV368	*Mycobacterium*	6
ASV90	*Nakamurella*	1
ASV515, ASV1081	*Nitrospira*	2
ASV40, ASV47, ASV66, ASV98, ASV169, ASV180, ASV271, ASV299	*Nocardioides*	8
ASV361	*Noviherbaspirillum*	1
ASV59	*Novosphingobium*	1
ASV267	*Oryzihumus*	1
ASV649	*Pedomicrobium*	1
ASV434	*Phenylobacterium*	1
ASV69	*Phyllobacterium*	1
ASV19	*Polaromonas*	1
ASV44, ASV65, ASV73, ASV461	*Pseudarthrobacter*	4
ASV52, ASV395	*Pseudolabrys*	2
ASV1, ASV2, ASV3, ASV5, ASV6, ASV8, ASV9, ASV13, ASV14, ASV16, ASV17, ASV46, ASV68, ASV76, ASV93, ASV156	*Pseudomonas*	16
ASV24, ASV28, ASV87, ASV165, ASV288, ASV289, ASV292, ASV625	*Pseudonocardia*	8
ASV383	*Pseudorhodoplanes*	1
ASV598	*Ramlibacter*	1
ASV315	*Reyranella*	1
ASV469	*Rhodococcus*	1
ASV105, ASV384, ASV402	*Rhodomicrobium*	3
ASV311, ASV444, ASV770	*Rhodoplanes*	3
ASV326, ASV932	*Rubrobacter*	2
ASV63, ASV312	*Saccharothrix*	2
ASV890	*Skermanella*	1
ASV75, ASV79, ASV293, ASV300, ASV404, ASV516	*Solirubrobacter*	6
ASV78, ASV89, ASV101, ASV103, ASV124, ASV144	*Sphingomonas*	6
ASV96	*Stenotrophomonas*	1
ASV382	*Steroidobacter*	1
ASV412, ASV552	*Streptomyces*	2
ASV15	*Tardiphaga*	1

ASV422	*TK10*	1
ASV401	*TRA3-20*	1
ASV22, ASV484	Frankiales	2
ASV57	Gemmatimonadaceae	1
ASV83, ASV285, ASV211, ASV294, ASV514, ASV42, ASV80, ASV314	Microbacteriaceae	8
ASV117, ASV227	Rhizobiales	2
ASV139, ASV257	Acetobacteraceae	2
ASV182, ASV304	Sutterellaceae	2
ASV185, ASV203, ASV266, ASV385	Beijerinckiaceae	4
ASV219, ASV301	Gaiellales	2
ASV273	Actinomarinales	1
ASV345	Sphingomonadaceae	1
ASV643, ASV842	Xanthobacteraceae	2
ASV889, ASV85, ASV575	Microtrichales	3
ASV10	*Variovorax*	1
ASV35	Stappiaceae	1
ASV54, ASV111	Sporichthyaceae	2
ASV192	Comamonadaceae	1
ASV193	Solirubrobacterales	1
ASV303	Caulobacteraceae	1
ASV305, ASV501	Oxalobacteraceae	2
ASV353	Propionibacteriaceae	1
ASV396	Xanthomonadaceae	1
ASV574	Micromonosporaceae	1
ASV624	Devosiaceae	1
ASV882	Rhizobiales	1
The ASVs shared between the rhizosphere soil and leaf endosphere		
ASV198	*0319-7L14*	1
ASV569	*67-14*	1
ASV81	*Actinoplanes*	1
ASV21, ASV297	*Allorhizobium-Neorhizobium-Pararhizobium-Rhizobium*	2
ASV284	*Amaricoccus*	1
ASV528	*Angustibacter*	1
ASV92, ASV399	*Arthrobacter*	2
ASV88, ASV120	*Bradyrhizobium*	2
ASV94, ASV155	*Brevundimonas*	2
ASV174	*Clavibacter*	1
ASV214	*Conexibacter*	1
ASV4, ASV77, ASV221	*Conyzicola*	3
ASV748	*Craurococcus-Caldovatus*	1
ASV30	*Devosia*	1
ASV95	*Escherichia-Shigella*	1
ASV23, ASV102, ASV482, ASV1051	*Gaiella*	4

ASV201	*Geodermatophilus*	1
ASV82, ASV108, ASV330, ASV421	*IMCC26256*	4
ASV38	*MB-A2-108*	1
ASV755	*Methylotenera*	1
ASV141	*Microbacterium*	1
ASV172	*Modestobacter*	1
ASV126	*Mycobacterium*	1
ASV636	*Myxococcus*	1
ASV90	*Nakamurella*	1
ASV40, ASV47, ASV66	*Nocardioides*	3
ASV238	*Oryzihumus*	1
ASV127	*Pedomicrobium*	1
ASV715	*Phycicoccus*	1
ASV69	*Phyllobacterium*	1
ASV44, ASV65, ASV73	*Pseudarthrobacter*	3
ASV395	*Pseudolabrys*	1
ASV1, ASV2, ASV3, ASV5, ASV6, ASV9, ASV13, ASV14, ASV16, ASV17, ASV76, ASV93, ASV143, ASV790	*Pseudomonas*	14
ASV290	*Pseudoxanthomonas*	1
ASV291	*Quadrisphaera*	1
ASV269	*Rhodococcus*	1
ASV618	*Romboutsia*	1
ASV932	*Rubrobacter*	1
ASV79	*Solirubrobacter*	1
ASV89, ASV101, ASV641, ASV756	*Sphingomonas*	4
ASV96	*Stenotrophomonas*	1
ASV337	*Sulfurifustis*	1
ASV394	*Sulfurirhabdus*	1
ASV757	*Thiobacillus*	1
ASV637, ASV670	*TK10*	2
ASV22, ASV152, ASV226	Frankiales	3
ASV57	Gemmatimonadaceae	1
ASV117	Rhizobiales	1
ASV139	Acetobacteraceae	1
ASV168	Gaiellales	1
ASV273	Actinomarinales	1
ASV285, ASV42, ASV509	Microbacteriaceae	3
ASV432, ASV274, ASV344	Solirubrobacteraceae	3
ASV10	Comamonadaceae	1
ASV35	Stappiaceae	1
ASV54	Sporichthyaceae	1
ASV80	Micrococcaceae	1
ASV85	Microtrichales	1
ASV230	Sphingomonadaceae	1
ASV322	Rhodobacteraceae	1

ASV353	Propionibacteriaceae	1
The ASVs shared between the root endosphere and leaf endosphere		
ASV198	*0319-7L14*	1
ASV118	*Acidovorax*	1
ASV21, ASV615	*Allorhizobium-Neorhizobium-Pararhizobium-Rhizobium*	2
ASV283	*Anaerococcus*	1
ASV88, ASV120	*Bradyrhizobium*	2
ASV94, ASV155, ASV158, ASV217	*Brevundimonas*	4
ASV251, ASV545, ASV872	*Chryseobacterium*	3
ASV174	*Clavibacter*	1
ASV481	*Conexibacter*	1
ASV4, ASV41	*Conyzicola*	2
ASV154, ASV616, ASV714, ASV833	*Corynebacterium*	4
ASV60, ASV67	*Cutibacterium*	2
ASV32	*Delftia*	1
ASV30	*Devosia*	1
ASV95, ASV145	*Escherichia-Shigella*	2
ASV23, ASV102	*Gaiella*	2
ASV1221	*Gemella*	1
ASV408	*Geodermatophilus*	1
ASV82, ASV108, ASV330	*IMCC26256*	3
ASV834	*Lactobacillus*	1
ASV352	*Lawsonella*	1
ASV259, ASV264, ASV393, ASV480	*Massilia*	4
ASV141	*Microbacterium*	1
ASV126	*Mycobacterium*	1
ASV90	*Nakamurella*	1
ASV40, ASV47, ASV66, ASV594, ASV669	*Nocardioides*	5
ASV130	*Noviherbaspirillum*	1
ASV712	*Pandoraea*	1
ASV468	*Pelagibacterium*	1
ASV565	*Peptoniphilus*	1
ASV69	*Phyllobacterium*	1
ASV44, ASV65, ASV73	*Pseudarthrobacter*	3
ASV395	*Pseudolabrys*	1
ASV1, ASV2, ASV3, ASV5, ASV6, ASV7, ASV9, ASV13, ASV14, ASV16, ASV17, ASV26, ASV39, ASV49, ASV72, ASV76, ASV93, ASV140	*Pseudomonas*	18
ASV932	*Rubrobacter*	1
ASV825	*Shinella*	1
ASV985	*SJA-28*	1
ASV79	*Solirubrobacter*	1
ASV89, ASV101, ASV265	*Sphingomonas*	3
ASV129, ASV619	*Staphylococcus*	2

ASV96	*Stenotrophomonas*	1
ASV747	*TK10*	1
ASV22	Frankiales	1
ASV57	Gemmatimonadaceae	1
ASV117	Rhizobiales	1
ASV139	Acetobacteraceae	1
ASV273	Actinomarinales	1
ASV285, ASV42	Microbacteriaceae	2
ASV10	*Variovorax*	1
ASV35	Stappiaceae	1
ASV54	Sporichthyaceae	1
ASV80	Micrococcaceae	1
ASV85	Microtrichales	1
ASV353	Propionibacteriaceae	1
ASV466	Hyphomicrobiaceae	1
ASV510	Enterobacteriaceae	1

Table S8. Key topological properties of bacterial co-occurrence networks, encompassing all taxa within the rhizosphere soil, leaf endosphere, and root endosphere.

Network properties							
Observed networks							
Edges	Nodes	Clustering coefficient	Average path length	Modularity	Graph density	Diameter	Average degree
947	230	0.48	3.69	0.45	0.04	12.48	8.23
Random networks							
		Clustering coefficient	Average path length		Modularity		
		0.036 ± 0.004	2.79 ± 0.007		0.31 ± 0.007		

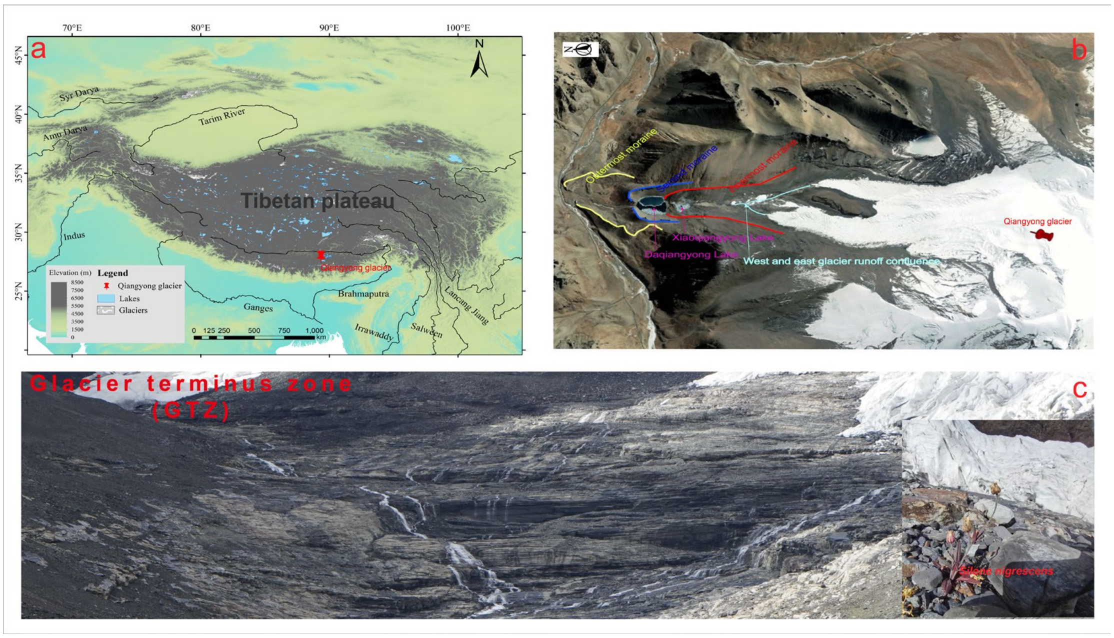

Figure S1. The location of the Qiangyong glacier (a, b), and the *Silene nigrescens* growing in the Qiangyong glacier terminus (c).

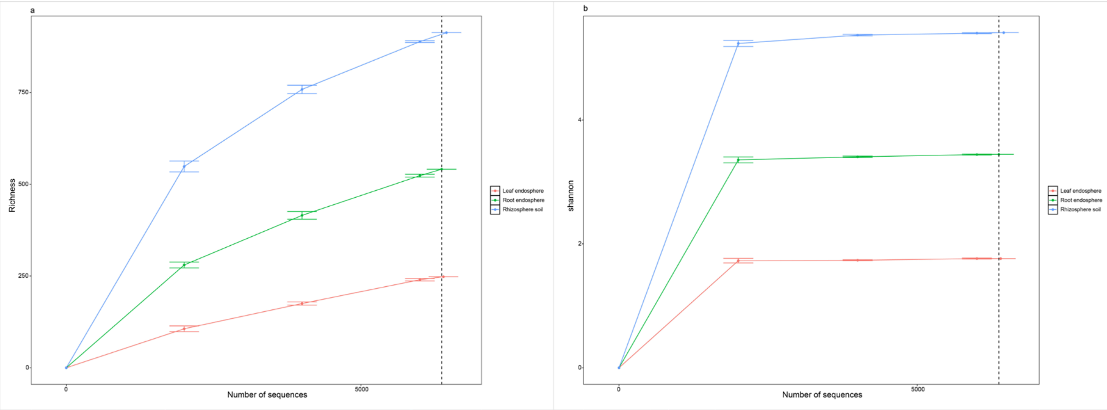

Figure S2. Rarefaction curves of ASV richness and Shannon diversity.
